# Supplementary material for: Endoglin Promotes Myofibroblast Differentiation and Extracellular Matrix Production in Diabetic Nephropathy
Source: Int J Mol Sci. 2020 Oct 18;21(20):7713. doi: 10.3390/ijms21207713 (PMC7589772; doi:10.3390/ijms21207713)
Supplement: Supplementary file 1 [file ijms-21-07713-s001.pdf]

Supplemental Table 1: Baseline characteristics of patients with histological confirmation of DN sorted by endoglin score

Characteristic			Eng <25% (n=20)	Eng 25%-50% (n=25)	Eng >50% (n=10)	*P*
Male sex		n (%)	12 (60.0)	17 (68.0)	5 (50.0)	0.601*
Age (years)		Mean ± SD	63.3 ± 16.9	70.2 ± 11.7	68.5 ± 10.3	0.101†
Type 1 Diabetes		n (%)	3 (17.6)	1 (4.3)	1 (12.5)	0.372*
eGFR (ml/min/1.73 m2)		Mean ± SD	60.3 ± 37.9	59.4 ± 33.2	21.3 ± 15.9	**0.007**†
Serum creatinine (µmol/L)		Mean ± SD	116.6 ± 69.4	137.1 ± 88.6	300.8 ± 129.8	**<0.001**†
Systolic blood pressure (mmHg)		Mean ± SD	133.5 ± 26.8	134.3 ± 30.9	168.9 ± 30.8	**0.049**†
Diastolic blood pressure (mmHg)		Mean ± SD	76.0 ± 15.1	76.0 ± 11.6	78.3 ± 6.5	0.823†
HbA1c (%)		Mean ± SD	7.2 ± 1.5	8.4 ± 2.2	9.3 ± 3.5	0.162†
Hypertension present		n (%)	5 (35.7)	15 (65.2)	6 (85.7)	**0.021***
IFTA	Index score 0	n (%)	8 (40)	1 (4)	0 (0)	
	Index score 1	n (%)	11 (55)	15 (60)	2 (20)	
	Index score 2	n (%)	1 (5)	7 (28)	3 (30)	
	Index score 3	n (%)	0 (0)	2 (8)	5 (50)	**<0.001***

*= calculated with a Chi-squared trent test; †= calculated with a spearman's rank correlation.
